# High “Normal” Blood Glucose Is Associated with Decreased Brain Volume and Cognitive Performance in the 60s: The PATH through Life Study

**DOI:** 10.1371/journal.pone.0073697

**Published:** 2013-09-04

**Authors:** Moyra E. Mortby, Andrew L. Janke, Kaarin J. Anstey, Perminder S. Sachdev, Nicolas Cherbuin

**Affiliations:** 1 Centre for Research on Ageing, Health and Wellbeing, The Australian National University, Canberra, Australia; 2 Centre for Advanced Imaging, The University of Queensland, Brisbane, Australia; 3 Neuropsychiatric Institute, University of New South Wales, Sydney, Australia; Charité University Medicine Berlin, Germany

## Abstract

**Context:**

Type 2 diabetes is associated with cerebral atrophy, cognitive impairment and dementia. We recently showed higher glucose levels in the normal range not to be free of adverse effects and to be associated with greater hippocampal and amygdalar atrophy in older community-dwelling individuals free of diabetes.

**Objective:**

This study aimed to determine whether blood glucose levels in the normal range (<6.1 mmol/L) were associated with cerebral volumes in structures other than the hippocampus and amygdale, and whether these glucose-related regional volumes were associated with cognitive performance.

**Design, Setting and Participants:**

210 cognitively healthy individuals (68–73 years) without diabetes, glucose intolerance or metabolic syndrome were assessed in the large, community-based Personality and Total Health Through Life (PATH) study.

**Main Outcome Measure:**

Baseline blood glucose levels in the normal range (3.2–6.1 mmol/l) were used to determine regional brain volumes and associated cognitive function at wave 3.

**Results:**

Higher blood glucose levels in the normal range were associated with lower grey/white matter regional volumes in the frontal cortices (middle frontal gyrus, inferior frontal gyrus precentral gyrus). Moreover, identified cerebral regions were associated with poorer cognitive performance and the structure-function associations were gender specific to men.

**Conclusion:**

These findings stress the need to re-evaluate what is considered as healthy blood glucose levels, and consider the role of higher normal blood glucose as a risk factor for cerebral health, cognitive function and dementia. A better lifetime management of blood glucose levels may contribute to improved cerebral and cognitive health in later life and possibly protect against dementia.

## Introduction

It is well established that type 2 diabetes is associated with ‘accelerated brain ageing’ [1], white matter lesions [2], atrophy [3], [4] and the presence of infarcts [5], which in turn relate to reduced cognitive functioning [1], [4], an increased risk of Alzheimer's disease [6], [7] and vascular damage [8]. A review by Awad et al. [9] of the relationship between impaired glucose tolerance, type 2 diabetes and cognitive function highlighted research linking sub-clinical levels of glucose in the high-normal range for glucose tolerance or impaired glucose tolerance (fasting glucose levels <7 mmol/L) with cognitive function, smaller hippocampal volumes and poor glucose regulation [10]. Notably, research has shown a decrement in cognitive function associated with impaired glucose tolerance in men, while women appear to demonstrate virtually identical scores to normoglycaemic women [11].

We have recently shown that higher glucose levels in the normal range (<6.1 mmol/L) are not necessarily free of adverse effects, and are associated with greater hippocampal and amygdalar atrophy in older community-dwelling individuals free of diabetes [12]. These findings are in accordance with animal studies demonstrating higher plasma glucose levels in rats to be associated with hippocampal neuronal loss, decreased neurogenesis, impaired spatial learning, reduced hippocampal dendritic spine density, and reduced long-term potentiation [13]–[15]. Furthermore, in non-diabetics, experimentally raised plasma glucose levels have been associated with increased systemic inflammation [16], [17], abnormal coagulation function [18], chronic stress and activation of the Hypothalamus-Pituitary-Adrenal axis [19], which are possible mechanisms that may explain these findings.

What is not known is whether glucose levels in the normal range are associated with cerebral volumes in structures other than the hippocampus and amygdala, and whether glucose-related regional volumes are associated with cognitive function. The aim of this study therefore was to assess whether blood glucose levels in the normal range (<6.1 mmol/L) are associated with volumes of other brain regions and to determine whether there is an association between these glucose-related regions and cognitive function in a large cohort of community-based individuals free of diabetes or cognitive impairment [20].

## Materials and Methods

### Ethics Statement

All participants gave written informed consent to be included in the Personality and Total Health (PATH) project. The study was approved by the Human Research Ethics Committee of The Australian National University.

### Subjects

Subjects were sampled from the Personality and Total Health Through Life (PATH) project, a large longitudinal study of ageing aimed at investigating the course of mood disorders, cognition, health and other individual characteristics across the lifespan [20]. PATH surveys 7485 individuals in three age groups of 20–24, 40–44 and 60–64 years at baseline. Follow-up is every four years over a period of 20 years. PATH surveys residents of the city of Canberra and the adjacent town of Queanbeyan, Australia, who were randomly recruited through the electoral roll [20]. Enrolment to vote is compulsory for Australian citizens, making this cohort representative of the population. The study was approved by the Australian National University Ethics Committee.

**Figure 1:**
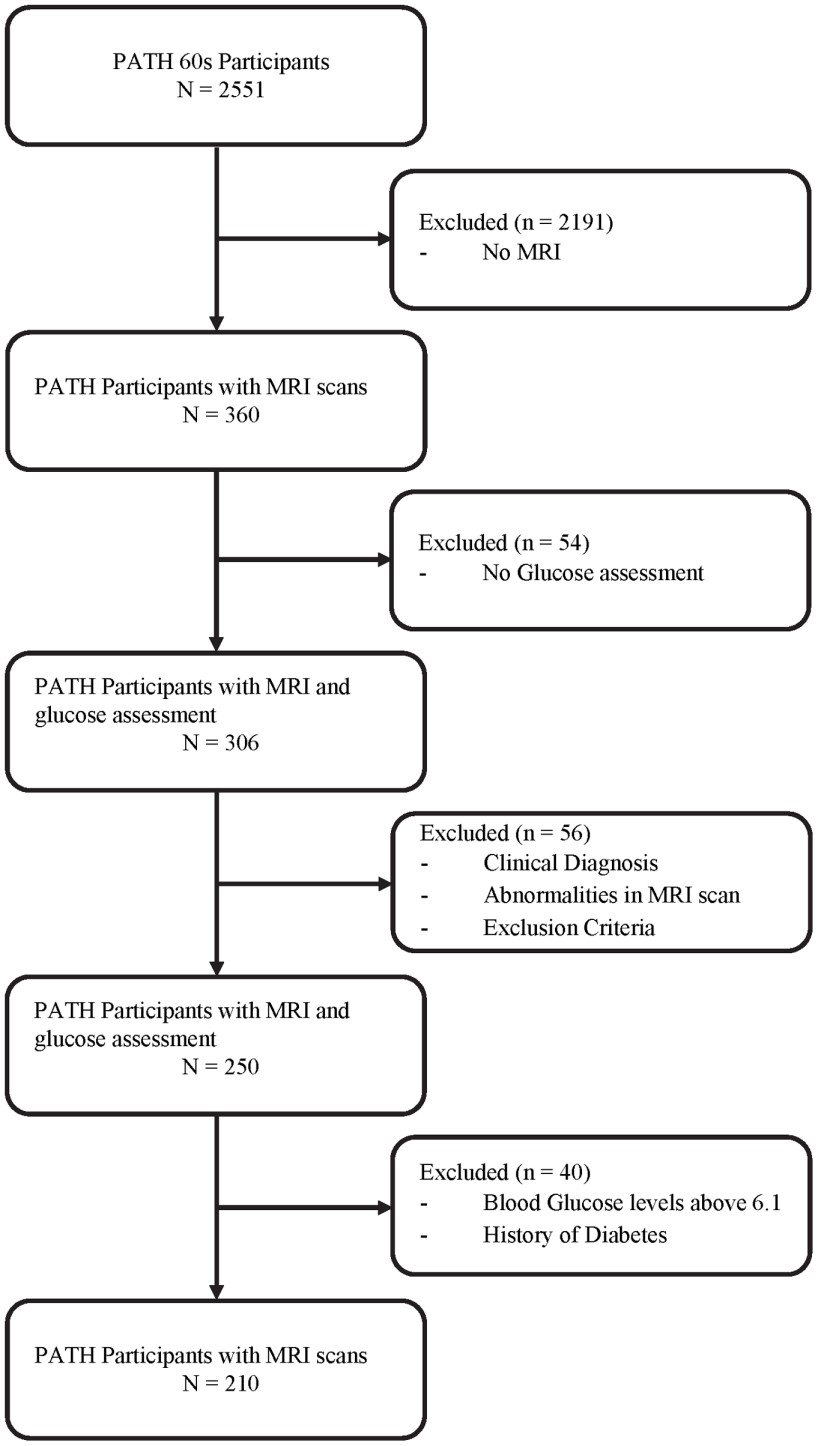
Sample Inclusion.

The present investigation is focused on the older participants (60 s cohort). Of the 2551 randomly selected older PATH subjects included in the study at wave 1, 2076 consented to be contacted regarding an MRI scan. Of these, a randomly selected subsample of 622 subjects was offered an MRI scan. 478 (77%; 252 men) eventually completed MRI scanning. Of these, 360 subjects (198 men; age range 68–74) were rescanned at wave 3. Of those with MRI scans, fifty-four (15%) were excluded from the current analyses as no blood glucose levels had been obtained at baseline. Fifty-six (18%) were excluded from the current analyses due to gross brain abnormalities (e.g. tumours, hydrocephalys; n = 18), a history of epilepsy, Parkinson's disease or stroke (n = 20), or a clinical diagnosis of any form of cognitive dysfunction (e.g. vascular dementia, mild cognitive impairment; n = 18). A further 40 subjects were excluded from the current analyses based on baseline blood glucose level above 6.1 mmol/L (110 mg/dl; n = 22) or a history of diabetes (n = 18) (Figure 1). A cut off of blood glucose levels above 6.1 mmol/L was used in accordance with the World Health Organisation threshold for impaired fasting hyperglycaemia (6.1 mmol/L, 110 mg/dl). This excluded individuals with diabetes, sub-clinical diabetes or glucose intolerance and ensured that the effect detected was not due to clinical or sub-clinical glucose metabolism abnormalities.

Following all exclusions, the final sample included 210 participants with glucose levels ranging between 3.2 and 6.1 mmol/l– a range considered to be representative of normal blood glucose levels. The final sample did not differ from the larger PATH sample on gender (χ^2^ (1, *N* = 2551)  = 0.139, *p* = 0.710), but had completed significantly more years of education (14.4 vs 13.7; *t* (2544) = −3.31, *p*<0.001).

### Socio-demographic and health measures

Socio-demographic information for race, years of education, alcohol consumption, smoking and depression were assessed using self-report. Body mass index (BMI) was based on subjects' self-report of weight and height and computed using the formula weight (kg)/height (m)^2^. The Alcohol Use Disorder Identification Test (AUDIT) was used to assess alcohol intake [21]. ‘For men, weekly alcohol consumption was categorized as light (1–13 units), moderate (14–27 units), hazardous (28–42 units) or harmful (>42 units). For women, weekly alcohol consumption was divided into light (1–7 units), moderate (8–13 units), hazardous (14–28 units) or harmful (>28 units) categories where a unit equates 10 g of pure alcohol’ [12].

### Blood glucose measures

Venous blood was collected at baseline following an overnight fast of at least 10 hours. Plasma and Serum aliquots were frozen at −80°C. Fasting plasma glucose was measured on a Beckman LX20 Analyzer by an oxygen rate method (Fullerton, California) [12].

### Neuropsychological tests

Cognitive performance was assessed at wave 3. All participants completed all cognitive assessments as part of the standardised procedure for test administration (same order of test administration for each participant). To avoid possible interaction effects between cognitive assessments the neuropsychological tests were separated by physical measure assessments (e.g. measuring of blood pressure, waist circumference or lung capacity).

Verbal working memory was assessed through the Digit Span Backwards (DSB) – a subtest of the Wechsler memory scale [22]. In the DSB participants were read numbers at one-second intervals. When participants incorrectly repeated both trials no further trials were given. Information and processing speed were assessed using the Symbol-Digit Modalities Test (SDMT) [23]. Participants were given 90 seconds to complete this task. Episodic memory was assessed using the first trial of the California Verbal Learning Test for both immediate (IRT) and delayed recall (DRT) [24]. Participants were read words at one second intervals and there was approximately one minute between immediate and delayed recall tests. Participants were not limited in the amount of time they could take to recall at either trial. The Boston Naming Test (BNT) [25] was used to assess language function. Participants were shown pictures of objects and asked to name the object. Executive function of verbal fluency was assessed using the Controlled Oral Word Association Test (COWAT) for both A- and F-words. Participants were timed for 60 seconds to list as many words starting with the letters A and F respectively.

### Data Acquisition

MRI scans used in this study were taken at wave 3. Subjects were scanned on a Siemens 1.5T Avanto scanner (Siemens Medical Solutions) for T1-weighted three-dimensional structural MRI. The T1 weighted MRI was acquired in sagittal orientation using the following parameters: repetition time (TR)/echo time (TE)  = 1.16/∼0.8 ms; flip angle  = 15°; matrix size  = 512×512; slice thickness  = 1.0 mm; resulting in a final voxel size of 1×0.5×0.5 mm.

### Image Processing

All images were pre-processed using the MINC imaging toolbox (MINC; http://en.wikibooks.org/wiki/MINC), images went through automatic QC to identify outliers via image histogram clamping and comparisons to the group minimum deformation average [26]. Images were then B0 MRI inhomogeneity corrected using N3 [27], and normalised via a linear correction to a global intensity model [26].

Optimized voxel-based morphometry (VBM) analyses were conducted using Statistical Parametric Mapping 8 (SPM8; Wellcome Department of Cognitive Neurology, London, UK, 2003) on Matlab 7.12 (Math Works, Natick, MA, USA, 2002). Images were first segmented into grey matter, white matter and cerebrospinal fluid [28]. Grey and white matter segmentations were further normalized to the sample template (population representative) which was generated by the diffeomorphic anatomical registration through exponentiated lie algebra (DARTEL) algorithm from participants' complete images [29]. DARTEL is a nonlinear warping technique that minimizes structural variation between subjects and has been evidenced to be more accurate than the standard normalization approach in SPM [30], [31]. Briefly, segmented images were registered, normalised and modulated to fit the DARTEL space, creating a DARTEL template based on the deformation fields produced in the segmentation procedure in which all individual deformation fields are warped (and modulated) to match this template [32]. Images were smoothed using a 8-mm, full-width-at-half-maximum Gaussian kernel to increase the signal-to-noise ratio, with each voxel of the resulting grey and white matter images representing the absolute amount of grey and white matter volume equivalent to their volume per unit before normalization [32].

### Image Data Analyses

Absolute total grey and white matter volumes were calculated using the native space grey and white matter segmentations. Smoothed grey and white matter density images were used in the voxel-wise regression analyses with fasting blood glucose levels as predictor. To account for the effects of possible confounding factors age, gender, BMI, depression and alcohol consumption were controlled for. Results were assessed at α = 0.0005.

### Statistical Analyses

Demographic characteristic analyses were conducted using IBM SPSS Statistics 20.0. After testing whether blood glucose levels in the normal range were associated with regional grey and white matter volumes, we next tested whether these glucose-related regional cluster volumes were associated with cognitive test performance. Grey and white matter regional volumes at significant voxels were extracted at the cluster level using the SPM8 “eigenvariate” extraction tool and standardized to Z-scores. In accordance with the literature [33], [34], and due to the nonparametric distribution of cognitive test performance, Spearman's Rank Order correlations was used to assess the association between the glucose-related regional volumes and cognitive test performance. Missing data for cognitive measures were imputed using the EM algorithm.

## Results

Participants' demographic characteristics are presented in Table 1.

**Table 1:** Wave 3 PATH Sample Descriptive.

Characteristics		Overall Sample (N = 210)	Male	Female	F/χ2
			(N = 111)	(N = 99)	male vs. female
**Age, years (SD)**		70.4 (1.42)	70.4 (1.42)	70.5 (1.42)	.231
** Range**		68–73	68–73	68–73	
**Race**					.456
** Caucasian, N (%)**		201 (95.7)	106 (95.5)	95 (95.9)	
** Asian, N (%)**		4 (1.9)	2 (1.8)	2 (2.02)	
** Other, N (%)**		3 (1.4)	1 (0.9)	2 (2.02)	
**Education, years (SD)**		14.4 (2.58)	15.3 (2.23)	13.4 (2.59)	31.9***
** Range**		5–19	11–19	5–19	
**MMSE, score (SD)**		29.4 (.876)	29.3 (.929)	29.5 (.800)	3.74
** Range**		26–30	26–30	26–30	
**BMI, score (SD)**		26.1 (4.74)	26.1 (3.53)	25.9 (5.82)	.028
	**Range**	18–60	20–37	18–60	
**Average Blood Glucose, level (SD)**		4.91 (.571)	4.99 (.509)	4.83 (.626)	4.15*
** Range**		3.2–6.1	3.4–6.0	3.2–6.1	
**Alcohol Consumption**					19.3**
** Abstain, N (%)**		13 (6.2)	5 (4.51)	8 (8.08)	
** Occasional, N (%)**		26 (12.4)	7 (6.31)	19 (19.2)	
** Light, N (%)**		110 (52.4)	72 (64.9)	38 (38.4)	
** Medium, N (%)**		45 (21.4)	18 (16.2)	27 (27.3)	
** Hazardous, N (%)**		12 (5.7)	7 (6.31)	5 (5.05)	
** Harmful, N (%)**		1 (0.5)	0 (0)	1 (1.01)	
**Smoke**					1.98
** History or Current, N (%)**		87 (41.4)	51 (45.9)	36 (36.4)	
**Depression**					.840
** No Depression, N (%)**		192 (91.4)	102 (91.9)	90 (90.9)	
** Subsyndromal Depression, N (%)**		12 (5.7)	5 (4.51	7 (7.07)	
** Minor Depression, N (%)**		2 (1.0)	1 (0.90)	1 (1.01)	
** Major Depression, N (%)**		3 (1.4)	2 (1.8)	1 (1.01)	
**Cognitive Test Performance**					
** Immediate Recall, mean (SD)**		7.06 (2.07)	6.64(1.77)	7.53 (2.27)	10.0**
** Range**		2–13	3–11	2–13	
** Delayed Recall, mean (SD)**		6.22 (2.23)	5.87 (1.93)	6.62 (2.47)	5.94**
** Range**		0–12	2–11	0–12	
** Digit Span Backwards, mean (SD)**		5.39 (2.07)	5.85 (2.04)	4.87 (1.99)	12.3***
** Range**		1–10	1–10	1–10	
** Symbol Digit Modalities mean (SD)**		49.9 (8.34)	49.9 (7.94)	49.9 (8.81)	.000
** Range**		16–71	27–66	16–71	
** COWAT F-words, mean (SD)**		14.5 (4.97)	15.0 (4.95)	13.9 (4.94)	2.29
** Range**		4–31	7–31	4–30	
** COWAT A-words, mean (SD)**		13.0 (5.45)	13.8 (5.35)	12.2 (5.47)	4.18*
** Range**		0–30	2–30	0–29	
** Boston Naming Test, mean (SD)**		13.8 (1.29)	13.9 (1.18)	13.7 (1.39)	2.09
** Range**		9–15	10–15	9–15	

* p<.05 ** p<.01 *** p<.001.

### Associations between fasting glucose levels and regional brain volumes

**Figure 2:**
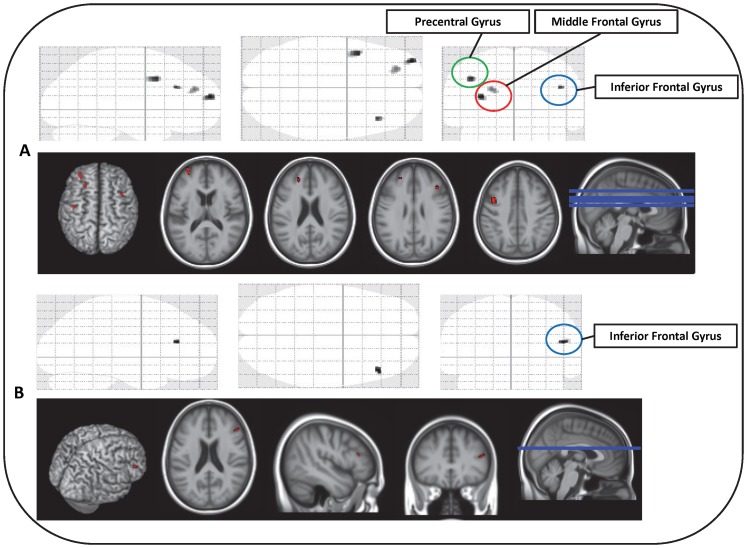
Sagittal, Coronal and Axial Representations of Glucose-related Regional Grey (A) and White (B) Matter Volumes.

Voxelwise analyses using continuous blood glucose levels as a predictor while controlling for age, gender, BMI, depression and alcohol consumption showed higher ‘normal’ plasma glucose levels to be associated with decreased regional grey (L Middle Frontal Gyrus, R Inferior Frontal Gyrus (Pars Triangularis), L Precentral Gyrus) and white matter (R Inferior Frontal Gyrus, Pars Triangularis) regional volumes (Table 2). Higher ‘normal’ blood glucose levels were notably associated with lower grey matter regional volumes in the left hemisphere and lower white matter regional volumes in the right hemisphere (Figure 2).

**Table 2:** Atlas Coordinates, Cluster Extents, P and T Values and Regional Descriptions.

	MNI coordinates (*x, y, z*)	Cluster extent (*k*)		Cluster-level *P* uncorrected	*T*	Region description (for cluster peak)
**Grey Matter**						
** Region 1**	−42, 8 39		113	<0.0005	3.85	Left Precentral Gyrus
** Region 2**	−22, 45, 25		59	<0.0005	3.63	Left Middle Frontal Gyrus
** Region 3**	42, 27, 28		20	<0.0005	3.77	Right Inferior Frontal Gyrus (Pars Triangularis)
** Region 4**	−33, 60, 16		66	<0.0005	3.97	Left Middle Frontal Gyrus
**White Matter**						
** Region 5**	48, 32, 19		21	<0.0005	3.80	Right Inferior Frontal Gyrus (Pars Triangularis)

### Volume-Cognition Relationships

Table 3 presents the associations between cognitive performance on individual tests and glucose-related regional grey/white matter volumes. Spearman's Rank Order correlations determined a significant positive association between grey matter regional volumes and a number of cognitive functions. Specifically, a positive correlation was found between the **Left Middle Frontal Gyrus (Region 2)** and raw scores on the DBS (*rs* = .158, *p* = .022), SDMT (*rs* = .207, *p* = .003), COWAT-F words (*rs* = .173, *p* = .012), COWAT-A words (*rs* = .172, *p* = .013) and BNT (*rs* = .173, *p* = .012). A positive correlation was also found between the **Right Inferior Frontal Gyrus (Region 3)** and raw scores on the DBS (*rs* = .193, *p* = .005). Furthermore, significant positive correlations were found between the **Left Middle Frontal Gyrus (Region 4)** and raw scores on the DBS (*rs* = .184, *p* = .007), and BNT (*rs* = .152, *p* = .027). It should be noted that a trend was also observed between the Left Middle Frontal Gyrus and performance on the COWAT-A words (*rs* = .135, *p* = .051). A further trend was also observed between white matter regional volumes of the **R Inferior Frontal Gyrus** (**Region 5)** and performance on the DBS (*rs* = .133, *p* = .054). No significant associations were found between cognitive performance and grey matter regional volumes in **L Precentral Gyrus (Region 1)**.

**Table 3:** The relationship between regional brain volumes and cognitive test performance.

Cognitive Test							
	IRT	DRT	DSB	SDMT	COWAT F	COWAT A	BNT
**Overall Sample**							
** Grey Matter**							
**Region 1** (L Precentral Gyrus)	.033	.016	.094	.057	.101	.046	.112
**Region 2** (L Middle Frontal Gyrus)	−.049	−.054	**.158** *	**.207** **	**.173** *	**.172** *	**.173** *
**Region 3** (R Inferior Frontal Gryus)	−.066	−.035	**.193** **	.069	.099	.097	.073
**Region 4** (L Middle Frontal Gyrus)	−.082	−.065	**.184** **	.066	.127	.135^1^	**.152** *
**White Matter**							
**Region 5** (R Inferior Frontal Gyrus)	−.043	−.085	.133^1^	.094	.032	.057	.044
**Male**							
**Grey Matter**							
**Region 1** (L Precentral Gyrus)	.158	.096	.078	−.024	.052	.052	.127
**Region 2** (L Middle Frontal Gyrus)	.159	.053	.079	**.256** **	**.202** *	.108	**.192** *
**Region 3** (R Inferior Frontal Gryus)	.047	.028	**.190** *	.154	**.207** *	**.198** *	.157
**Region 4** (L Middle Frontal Gyrus)	**.222** *	.171	.156	.098	.164	.140	**.190** *
**White Matter**							
**Region 5** (R Inferior Frontal Gyrus)	.080	−.044	.010	.097	.172	.108	.084
**Female**							
**Grey Matter**							
**Region 1** (L Precentral Gyrus)	.053	.060	−.051	.166	.122	−.024	.059
**Region 2** (L Middle Frontal Gyrus)	.020	.045	−.006	**.248** *	.160	.165	.114
**Region 3** (R Inferior Gryus)	−.016	.004	.046	.018	.003	−.026	−.065
**Region 4** (L Middle Frontal Gyrus)	−.116	−.121	−.014	.071	.024	−.002	.057
**White Matter**							
**Region 5** (R Inferior Frontal Gyrus)	−.005	−.038	.083	.078	−.192^2^	−.085	−.094

Abbreviations: IRT: California Verbal Learning Test for Immediate Recall, DRT: California Verbal Learning Test for Delayed Recall, DSB: Digit Span Backwards from the Wechsler Memory Scale, SDMT: Symbol-Digit Modalities Test, COWAT-A/COWAT-F Controlled Oral Word Association Test for A- and F-words, BNT: Boston Naming Test.

^1^ p-value approaching significance (i.e. p = .054 and p = .051).

^2^ p-value approaching significance (i.e. p = .056).

** p<.01.

* p<.05.

Interestingly, this study found the association between grey matter regional volumes and cognitive function to be gender specific. In men, small to medium effects were found between grey matter regional volumes in the **Left Middle Frontal Gyrus (Region 2)** and raw scores on the SDMT (rs = .256, p = .007), COWAT-F words (*rs* = .202, *p* = .033), and BNT (*rs* = .192, *p* = .043); between the **Right Inferior Frontal Gyrus (Region 3)** and DBS (rs  = .190, p = .046), COWAT-F words (*rs* = .207, *p* = .030), and COWAT-A words (rs = .198, p = .037). Small to medium effects were also found between grey matter regional volumes of the **Left Middle Frontal Gyrus (Region 4)** and raw scores on the IRT (rs = .222, p = .019), and BNT (rs = .190, p = .045). In men, white matter regional volumes of the **R Middle Frontal Gyrus (Region 5)** were not associated with cognitive performance.

In women, grey matter regional volumes in the **Left Middle Frontal Gyrus (Region 2)** were only associated with raw scores on the SDMT (*rs* = .248, *p* = .013). Notably, in women, a negative trend was observed between the Right Inferior Frontal Gyrus and performance on the COWAT-F words (*rs* = −.192, *p* = .056).

### Sensitivity Analyses

To determine whether the observed effects of normal blood glucose levels are modulated by diabetes, analyses were also conducted with all participants with a blood glucose level in the normal range (<6.1 mmol/L), including those reporting a history of diabetes (n = 231). The results followed the same pattern as that presented above.

## Discussion

This study investigated the association between normal blood glucose levels and grey and white matter regional volumes across the whole brain and its association with cognitive performance in a large, community-based sample of cognitively healthy individuals without diabetes, glucose intolerance or metabolic syndrome. Three important findings were made: 1) higher glucose levels in the normal range were associated with lower grey and white matter regional volumes; 2) lower glucose-related grey/white matter regional volumes were associated with poorer cognitive performance; 3) structure-function associations were gender specific.

In voxel-wise analyses high-normal blood glucose levels were associated with lower grey and white matter regional volumes in the frontal cortices – areas associated with increased age-related decline and episodic memory impairments (e.g. executive function) [35], [36]. In particular, high-normal blood glucose levels were found to be associated with lower regional grey matter volumes in the left middle frontal gyrus, right inferior frontal gyrus (Pars Triangularis) and the left precentral gyrus and lower regional white matter regional volumes in the right inferior frontal gyrus. Notably, the predominant left hemisphere findings observed in relation to higher-normal blood glucose levels in our study are consistent with previous research demonstrating higher left hemisphere vulnerability in pathological ageing [37]. In Alzheimer's disease, frontal regions are disproportionately atrophic (after brain size correction) with greater left versus right hemisphere atrophy observed [38]. These findings are of particular interest as evidence suggests that diabetes is associated with greater amyloid plaque deposition and greater cerebro-vascular damage, which forms the anatomical basis for clinical and subclinical cognitive impairment [6], [8]. The present findings suggest a similar pattern of lower grey/white matter regional volumes in the pre-frontal and frontal cortices consistent with those found in individuals prior to the onset of dementia [38].

Importantly, this study found that smaller regional volumes associated with high-normal blood glucose levels were linked to poorer cognitive performance. Specifically, smaller regional volumes appeared to be associated with poorer working memory, executive functions, information and processing speed, and language function. These findings are in accordance with literature linking both subclinical [9] and diabetic glucose levels [39] to cognitive performance but extend them by showing that this association is also present in what is considered plasma glucose's normal range (up to 6.1 mmol/L). Future research should consider the possible impact of these findings in terms of glucoregulation in the ‘higher-normal’ range and its relationship with memory performance, as increased metabolic demand during regional activation (i.e. memory testing) may be associated with regional low-grade hypoglycaemia and ultimately an inability to compensate for drops in glucose levels during the activation of these circuits during memory testing [10].

Finally, the association between glucose-related regional volumes and cognitive test performance were gender specific. For women, only smaller grey matter regional volumes of the left middle frontal gyrus were significantly associated with information and processing speed, a process shown to be impaired in women with type 2 diabetes [39]. However, for men, smaller grey matter regional volumes were significantly associated with poorer information and processing speed, episodic memory, and executive function, while smaller white matter regional volumes were associated with poorer language function. These findings raise interesting questions regarding gender specific impact of normal blood glucose levels on cerebral structure and cognitive performance in healthy individuals in their 60 s. Research has shown the incidence of Alzheimer's disease to be consistently higher in women and the incidence of Vascular Dementia to be consistently higher in men [40]. Such gender differences may be due to biological, survival or cohort differences in the exposure to protective or risk factors [40]. The protective role of estrogens, cardiovascular disease and survival rates may provide an explanation for the current findings. A better understanding of the impact of higher blood glucose levels in the normal range on cardiovascular disease in men may provide further insight into the gender-specific association of grey and white matter regional volumes and cognitive performance in the current study.

It is important to consider possible mechanisms which may underpin the association between blood glucose levels, vascular variations, regional volumes (e.g. atrophy) and cognitive change. Possible mechanisms proposed to be involved include inflammatory processes [12], as research has linked experimentally raised glucose levels with increased plasma cytokine levels (tumor necrosis factor-α, interleukin-6 and interleukin-10) in healthy individuals and those with impaired glucose tolerance [16]. Furthermore, this study by Esposito et al. [16] found that inflammatory reactions peaked higher and lasted longer in IGT subjects, indicating that control of plasma glucose levels modulates systemic inflammatory responses and that individuals with type 2 diabetes are therefore more likely to be exposed to longer and stronger inflammatory states [12]. As chronic systemic inflammation has been linked to cerebral atrophy, it is therefore also plausible that blood glucose levels in the higher normal range may also be associated with neurodegeneration [12]. The current findings provide further evidence of this association.

Furthermore, research has linked coagulation-related abnormalities to type 2 diabetes (i.e. raised tissue factor, factor VII, (pro-)thrombin, fibrinogen levels) [41]. Darvall and colleagues [42] have linked such alterations to abnormal clotting and greater cardiovascular events and the activation of prothrombic pathways have also been associated with poorly regulated blood glucose levels in prediabetic states (e.g. insulin resistance, metabolic syndrome) [12]. As prediabetic states (e.g. metabolic syndrome, insulin resistance) have been linked to an increased risk of thrombosis [43], it is therefore possible that high glucose levels in the absence of type 2 diabetes may result in abnormal coagulation function, therefore increasing the risk of thrombosis, microemboli and clinical and subclinical strokes – all which are known risk factors of cerebral brain ageing [12].

It should also be noted that psychological stressors have also been associated with an higher risk of type 2 diabetes, with depressive individuals having been found to have a 60% increased risk of diabetes [44]. As noted, chronic stress is associated with increased hypothalamic-pituitary-adrenal (HPA) axis activation, which in turn is also associated with higher glucose levels [19]. It is therefore plausible that stressors may contribute to higher glucose levels and associated brain effects [12]. While not focused on the mechanisms underpinning the association between glucose levels, regional brain volumes and cognition, our findings contribute to the existing understanding and provide further evidence for an association between what is considered to be ‘normal’ blood glucose levels, cerebral integrity and cognition.

This study had a number of limitations but also several strengths. First, while PATH is a population representative study in which participants were randomly selected into the neuroimaging sub-study from the larger population-representative cohort, it may not be completely representative of the population at large. Moreover, the selection procedure which excluded those with pre-clinical or diagnosed diabetes, neuro-cognitive disorders and brain abnormalities further decreased the representativeness of the sample. However, the overall effect of this methodology was to select overall healthier individuals and therefore any effect detected in this study is likely to be an underestimate of those applying to the general population. Secondly, while the use of a narrow age cohort avoids some biases due to cohort effect and therefore allows for more sensitive detection of associations between normal blood glucose, structural brain volumes and cognitive performance, it may also make it more difficult to generalize the current findings to other age groups. Thirdly, although a widely accepted WHO cut-off level for normal blood glucose (<6.1 mmol/L) was used, it must be noted that cut-offs for normality are being debated and the American Diabetes Association has already adopted a normal fasting level of <5.6 mmol/L. Fourthly, the assessment of plasma glucose at solely one time point provides a further limitation of this study. As plasma glucose levels are known to fluctuate, this single observation of glucose levels may not be truly representative of the on-going glucose state of an individual. Future studies, where possible, should include assessments of HBA1c levels – a measure of average blood glucose control over 2–3 months – to obtain a more representative measure. Finally, it must be noted that while blood glucose levels were assessed following a minimum of 10 hours fasting, there are many factors which may contribute to variations in glucose levels. For example, it is known that blood glucose levels progressively increase across the adult lifespan and that cognition tends to decrease with age, especially in old age. Therefore, associations between blood glucose and cognition may be detected not due to direct associations but due to their association with age. To minimise possible confounding effect of age, the current study used a narrow age cohort design (4 year age range) and also controlled for age in the statistical analyses. It is therefore unlikely that age effects explain the findings reported in the current study. BMI is also a likely confounding factor as obesity is associated with increased caloric intake, higher risk of insulin resistance, greater cerebral atrophy and cognitive decline. To minimise possible confounding effects, the current study controlled for BMI. However, effects due to BMI may not have been completely accounted for and could potentially explain some of our findings. Further, it is also known that depression contributes to poorer cognition in diabetes. While this study controlled for depression to minimise possible confounding effects, it is possible that effects due to depression may not have been completely accounted for and that these could potentially also explain some of our findings. Other factors which are potential confounders, but which are less relevant to the studied population, include medications (e.g. corticosteroids, diuretics, epinephrine, estrogen, glucagon, lithium and antidepressants), medical conditions (e.g. chronic renal failure, Cushing's syndrome, over or under active thyroid, pancreatic cancer, adrenal insufficiency, liver disease, underactive pituitary gland), activation of the stress response, the type and quantities of food eaten, and drug use (e.g. acetaminophen, alcohol, anabolic steroids, monoamine oxidase inhibitors and pentamidine). Notably, these confounds may not only impact the accuracy of the glucose readings, but may also independently impact brain structure and cognitive function.

Despite these limitations, this study is characterized by significant strengths. First, its large sample size makes this study significantly more representative of the population than others described in the diabetic literature (mainly self-selected and clinical samples). Secondly, the exclusion of individuals with diabetes, sub-clinical diabetes or glucose intolerance based on the World Health Organisation threshold for impaired fasting hyperglycaemia (6.1 mmol/L, 110 mg/dl) ensured that the effects detected were not due to clinical or sub-clinical glucose metabolism abnormalities. Thirdly, to ensure the effects detected related to normal blood glucose levels, individuals with other neurological disorders were excluded. This allowed for the investigation of long term associations between normal blood glucose levels, brain structure and cognitive performance despite a cross-sectional design. In conclusion, the current findings stress the need to re-evaluate what is considered healthy blood glucose levels, investigate possible mechanisms contributing to a possible modulating effect between normal blood glucose levels and pathological change, and consider the role of higher normal blood glucose levels as a risk factor for cerebral health, cognitive function and dementia. A better lifetime management of blood glucose levels may ultimately contribute to improved old age memory and possibly have a protective effect against cognitive impairments.
